# Effect of Low Temperature on Insecticidal Protein Contents of Cotton (*Gossypium herbaceum* L.) in the Boll Shell and Its Physiological Mechanism

**DOI:** 10.3390/plants12091767

**Published:** 2023-04-26

**Authors:** Zhenyu Liu, Mingyu Ji, Run He, Yuyang Dai, Yuting Liu, Nana Mou, Jianing Du, Xiang Zhang, Dehua Chen, Yuan Chen

**Affiliations:** Jiangsu Key Laboratory of Crop Genetics and Physiology, Co-Innovation Center for Modern Production Technology of Grain Crops, Yangzhou University, Yangzhou 225009, China

**Keywords:** Bt cotton, boll shell, insecticidal protein, low temperature, nitrogen metabolism

## Abstract

Low temperature is the main factor for global natural disasters affecting the growth and distribution of plants, and cotton may be affected by low temperature and cold damage at all growth stages. In addition, the insecticidal resistance of cultivars has been reported to perform poorly or unstably due to adverse environments. The present study aimed to investigate the impact of low temperature on the levels of insecticidal protein in *Bacillus thuringiensis* (Bt) transgenic cotton plants during the peak boll stage. To achieve this, two Bt cotton cultivars, Sikang1 (SK1) and Sikang3 (SK3), were subjected to different temperature regimes and durations. The findings of the study demonstrated that the expression of insecticidal protein in the boll shell of Bt transgenic cotton plants was significantly inhibited under low-temperature stress. Specifically, in 2020, compared to the CK (27 °C), the insecticidal protein content in the boll shell of SK3 decreased by 28.19% after a 48 h of a 16 °C temperature. These results suggest that low-temperature stress can negatively impact the expression of insecticidal protein in Bt transgenic cotton, highlighting the need for appropriate measures to minimize its adverse effects on cotton production. In addition, the threshold temperature that leads to a significant decrease in the content of insecticidal proteins symbolizes an upward trend as the duration of stress prolongs. Decreased Bt protein content at low temperatures is associated with changes in the N metabolism. The present study revealed a significant positive correlation between the levels of glutamate oxaloacetate transaminase (GOT) and glutamate pyruvate transaminase (GPT) activities, as well as in the soluble protein levels in the boll shell and the content of the Bt protein. On the other hand, a significant negative correlation was observed between the levels of free amino acids, peptidase, and protease activities, as well as of Bt protein content. These findings suggest that, in Bt cotton production, it is crucial to remain vigilant of prolonged low-temperature disasters, which last for over 12 h and drop below 17–20 °C during the peak boll stage. Such conditions may reduce insecticidal resistance, leading to substantial economic losses.

## 1. Introduction

The insecticidal protein produced by *Bacillus thuringiensis* (Bt) can destroy the intestinal cells of lepidopterans, causing imbalance in cell osmosis and ultimately killing insects [1,2,3]. Before the emergence of transgenic insect-resistant cotton, Bt emulsion, as a biological insecticide, was widely used in agricultural production because of its strong specificity and safety for humans and animals [4]. The research of genetically modified cotton began in the 1980s of the 20th century, and, in 1995, China had obtained a number of insect-resistant cotton strains, whose insect resistance effect and yield were superior to the introduced Bt cotton carrying the Cry gene [5,6]. Since 1997, the extensive cultivation of Bt cotton has brought high economic and ecological benefits to China: it has reduced the harm of lepidopteran pests—such as bollworm—to cotton, reduced the economic loss caused by the reduction in cotton production, reduced the use of pesticides, and reduced damage to the environment [7,8,9,10]. Due to the unstable expression of Bt cotton insect resistance, the insecticidal effect is only about 40%, so related pests such as bollworm are still the main pests affecting cotton production [11,12]. The main reason for this is that the expression of Bt insecticidal protein (Bt protein) is characterized by spatiotemporal distribution [11,13,14]. In terms of time, the amount of Bt protein in the seedling bud stage is high, and it weakens after flowering. In terms of space, the expression of Bt protein in different organs and different growth stages of the same organ is different, and the overall expression is high in leaves and low in reproductive organs, especially regarding the lowest expression of Bt protein in cotton bolls in the boll period. Moreover, the most serious period of damage to cotton by bollworm is also in the boll stage [11,15,16,17].

Environmental factors have a significant influence on the expression of Bt cotton insecticidal protein. When the light of the Bt cotton plant is not sufficient for a long period of time, the middle and lower leaves gradually age, the upper young leaves are born slowly, and the ability of the cotton plant to synthesize Bt protein decreases. Abel et al. [18] also obtained similar results by studying the insect resistance of cotton boll. They analyzed the reasons for this, and concluded that it may be due to the cover of wilting petals and other substances, the tip of the boll shell being less exposed to sunlight, and the normal level of chlorophyll not being able to be synthesized, so the low content of Bt protein leads to reduced insect resistance. Temperature and moisture will affect the expression of Bt cotton insect resistance [19,20,21]. Lu et al. [22] studied the effect of diurnal temperature change stress on the insect resistance of Bt cotton, and concluded that extreme high temperature during the day and extreme low temperature at night can significantly affect the insect resistance of Bt cotton. The longer the stress time, the stronger the impact. Liu and Chen et al. [23,24] also came up with similar results, and also concluded that the Bt protein content in plants can return to normal levels within a certain period of time after the stress is terminated. Zhang et al. [25] concluded that, under the stress of water deficiency, protein synthesis is weakened and decomposition is strengthened, resulting in a decrease in Bt protein content in bell shells. Extreme temperatures and humidity can cause a decrease in the content of Bt cotton insecticidal protein [26,27]. Soil water content and soil temperature significantly affect the degradation of Bt protein in soil [28]. Thus, investigating the impact of unfavorable environmental conditions on the production of insecticidal protein in Bt cotton has emerged as a critical aspect of enhancing insect resistance.

Cotton is a temperature-loving crop and is sensitive to low temperatures. As China’s cotton planting areas gradually shift to cold and cool areas, such as in Xinjiang and Inner Mongolia, low-temperature stress will accompany the whole growth period of cotton. Shi et al. [29] found that low temperature duration is the dominant factor affecting the germination rate of cotton seeds. Li et al. [30] found that at low-temperature levels of 5 °C, 10 °C, and 15 °C, with the increase in treatment time, the structure and morphology of cotton seedlings changed, the dry matter accumulation decreased, the cell membrane permeability increased, and weak seedlings were formed. Under the same duration and low-temperature stress, the stem thickness and relative conductivity of cotton seedlings changed significantly. The research conducted by Chen et al. [26] substantiates that low-temperature stress has a more notable impact on the synthesis of *Bacillus thuringiensis* (Bt) insecticidal protein than high-temperature stress. Despite the multitude of investigations that have been conducted on the impact of low temperature on cotton, a significant gap in comprehensive research still exists regarding the influence of varying levels and durations of low temperature on the expression of Bt insecticidal protein. Prior studies have predominantly focused on cotton seeds or cotton squares [31,32,33,34], with only limited attention given to the reproductive organs primarily affected by bollworms, particularly the cotton boll. Current research findings suggest that the levels of Bt insecticidal protein in reproductive structures, including squares and bolls, are substantially lower in comparison to those in leaves. Given that the cotton boll is a crucial food source for cotton bollworms and a primary target for yield in cotton cultivation, investigation into the impact of low temperatures on insect resistance in cotton production is imperative. Therefore, the objective of this study is twofold: (1) to examine the influence of low-temperature levels and stress duration on the expression of Bt insecticidal protein in the boll shell, and (2) to elucidate the underlying mechanisms that mediate the effect of low temperatures on the expression of Bt insecticidal protein in the boll shell.

## 2. Results

### 2.1. Low-Temperature Effect on the Insecticidal Protein Content of the Boll Shell

In comparison to the control temperature of 27 °C, low-temperature exposure elicited a reduction in the levels of insecticidal protein in the boll shell of Bt transgenic cotton plants (Table 1). The content of insecticidal protein demonstrated a corresponding downward trend with a decreasing temperature. In the year 2020, a substantial reduction in Bt protein content was observed in the cultivars Sikang1 (SK1) and Sikang3 (SK3) following 12 h of low-temperature treatment at 16 °C and 17 °C when compared to the optimal temperature. Following exposure to low-temperature stress, a decrease in the insecticidal protein levels was observed in the boll shell of Bt transgenic cotton plants in cultivars SK1 and SK3. The reduction was significant at temperatures of 16 °C, 17 °C, and 18 °C after 24 h of exposure, and at temperatures of 16 °C, 17 °C, 18 °C, 19 °C, and 20 °C following 48 h of exposure. The critical low-temperature thresholds for a significant decrease in insecticidal protein content were found to be 17 °C after 12 h of stress, 18 °C after 24 h of stress, and 20 °C after 48 h of stress for SK1 and SK3. Comparable results were observed in 2021, with the threshold temperatures being 17 °C, 18 °C, and 19 °C after 12, 24, and 48 h of low-temperature stress in SK1, and 17 °C, 18 °C, and 18 °C after 12, 24, and 48 h of low-temperature stress in SK3, respectively. The results indicate that prolonged low-temperature stress led to an increase in the threshold temperature and a further decrease in the insecticidal protein levels compared to the control.

The impact of low temperature on the Bt protein content in cotton boll shells was examined in 2020 and 2021. After a 12 h low-temperature treatment in 2020, the Bt protein content decreased by 0.71–11.17% in SK1 and 0.48–7.05% in SK3, at temperatures ranging from 16 °C to 20 °C. After 24 h, the content decreased by 4.15–23.83% in SK1 and 3.62–23.59% in SK3, while after 48 h, it decreased by 7.72–27.66% in SK1 and 8.22–28.19% in SK3. Similar results were obtained in 2021. The extension of the duration of low-temperature stress resulted in a heightened threshold temperature and a further reduction in the Bt protein content in comparison to the control.

### 2.2. Low Temperature on the Nitrogen Metabolism of the Boll Shell

**Figure 1:**
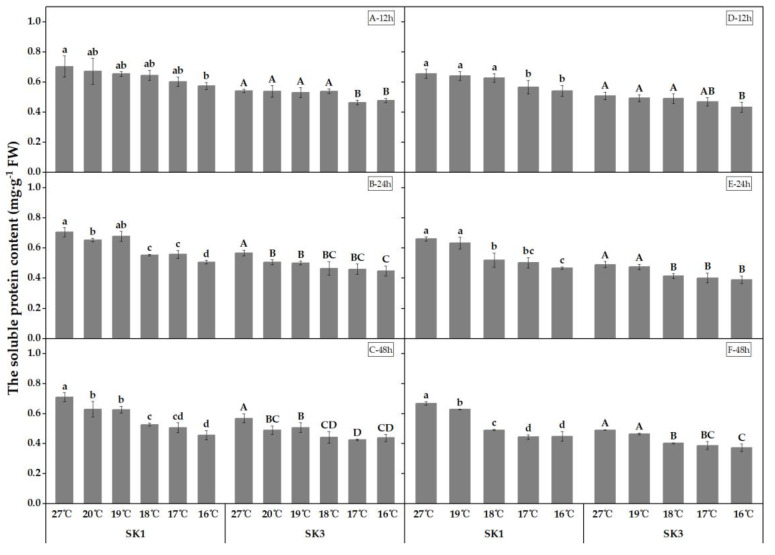
Effect of low-temperature stress on soluble protein content in boll shells in 2020 (A−12 h stress; B−24 h stress; and C−48 h stress) and 2021 (D−12 h stress; E−24 h stress; and F−48 h stress). Statistical analysis showed that there were significant differences between the treatments of the SK1 variety when represented by different lowercase letters, and between the treatments of the SK3 variety when represented by different capital letters (*p* < 0.05).

The effect of low temperature on the soluble protein content in cotton boll shells was examined in the present study. As shown in Figure 1, a decrease in soluble protein content was observed as the temperature decreased. Prolonged exposure to low temperature resulted in an elevation of the threshold temperature required for a significant reduction in soluble protein content. In 2020, SK1 showed a threshold temperature of 16 °C, 18 °C, and 20 °C after 12, 24, and 48 h of low-temperature treatment, respectively, while SK3 showed a threshold temperature of 17 °C, 20 °C, and 20 °C under the same conditions. Similarly, in 2021, SK1 exhibited a threshold temperature of 17 °C, 18 °C, and 19 °C after 12, 24, and 48 h of low-temperature treatment, respectively, while SK3 showed a threshold temperature of 16 °C, 18 °C, and 18 °C under the same conditions.

In 2020, after 12 h of low-temperature treatment, soluble protein contents decreased by 18.44%, 14.34%, 8.42%, 7.04%, and 4.56% in SK1, and by 11.85%, 14.18%, 0.37%, 2.13%, and 0.73% in SK3 at 16 °C, 17 °C, 18 °C, 19 °C, and 20 °C, respectively. After 24 h of low-temperature treatment, soluble protein contents decreased by 28.06%, 20.89%, 21.49%, 3.86%, and 7.28% in SK1, and by 20.91%, 19.12%, 18.19%, 11.70%, and 10.92% in SK3 at 16 °C, 17 °C, 18 °C, 19 °C, and 20 °C, respectively. After 48 h of low-temperature treatment, soluble protein contents decreased by 35.52%, 28.50%, 25.64%, 11.59%, and 11.19% in SK1, and by 22.86%, 25.17%, 22.30%, 10.67%, and 13.69% in SK3 at 16 °C, 17 °C, 18 °C, 19 °C, and 20 °C, respectively. A similar trend was observed in 2021. These findings indicate that an extension of the low-temperature stress treatment duration led to an increase in the threshold temperature and to a further decline in the concentration of soluble protein in the boll shell relative to the control group.

**Figure 2:**
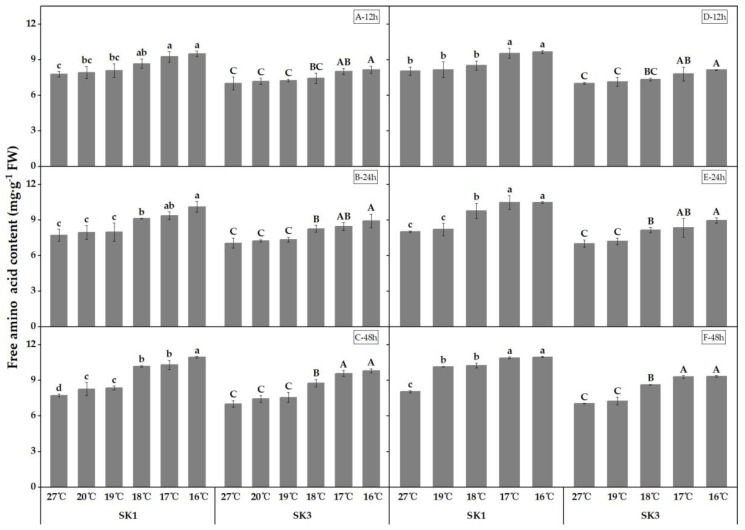
Effect of low-temperature stress on free amino acid content in boll shells in 2020 (A−12 h stress; B−24 h stress; and C−48 h stress) and 2021 (D−12 h stress; E−24 h stress; and F−48 h stress). Statistical analysis showed that there were significant differences between the treatments of the SK1 variety when represented by different lowercase letters, and between the treatments of the SK3 variety when represented by different capital letters (*p* < 0.05).

In contrast to the decreased content of soluble protein, the findings of this study revealed an increase in free amino acid content as a response to low-temperature treatment, as demonstrated in Figure 2. Notably, with an extended duration of exposure to low temperature, the threshold temperature causing a significant elevation in free amino acid content in the boll shell was observed to increase. Specifically, in 2020, the threshold low temperature for SK1 was found to be 18 °C, 18 °C, and 20 °C after low-temperature treatment for 12 h, 24 h, and 48 h, respectively, while for SK3, the corresponding threshold low temperature was 17 °C, 18 °C, and 18 °C. In 2021, the threshold temperature for SK1 was determined to be 17 °C, 18 °C, and 19 °C after 12 h, 24 h, and 48 h of low-temperature stress, while for SK3, it was found to be 17 °C, 18 °C, and 18 °C.

In 2020, after 12 h of low-temperature treatment, the free amino acid contents increased by 11.66%, 19.15%, 22.15%, 4.21%, and 2.08% in SK1 and 6.24%, 14.50%, 16.44%, 3.51%, and 2.61% in SK3 at 16 °C, 17 °C, 18 °C, 19 °C, and 20 °C, respectively. After 24 h of low-temperature treatment, the free amino acid contents increased by 18.27%, 21.37%, 31.10%, 3.51%, and 3.06% in SK1 and 17.09%, 19.74%, 26.60%, 4.23%, and 2.55% in SK3 at 16 °C, 17 °C, 18 °C, 19 °C, and 20 °C, respectively. After 48 h of low-temperature treatment, the free amino acid contents increased by 31.84%, 33.40%, 41.70%, 8.20%, and 7.07% in SK1 and 24.71%, 36.44%, 39.65%, 7.58%, and 5.83% in SK3 at 16 °C, 17 °C, 18 °C, 19 °C, and 20 °C, respectively. The same trend was observed in 2021.

The results indicated that an extension of the low-temperature stress treatment duration not only raised the critical low temperature, but also led to an elevation in the free amino acid contents in the boll shell relative to the control.

**Figure 3:**
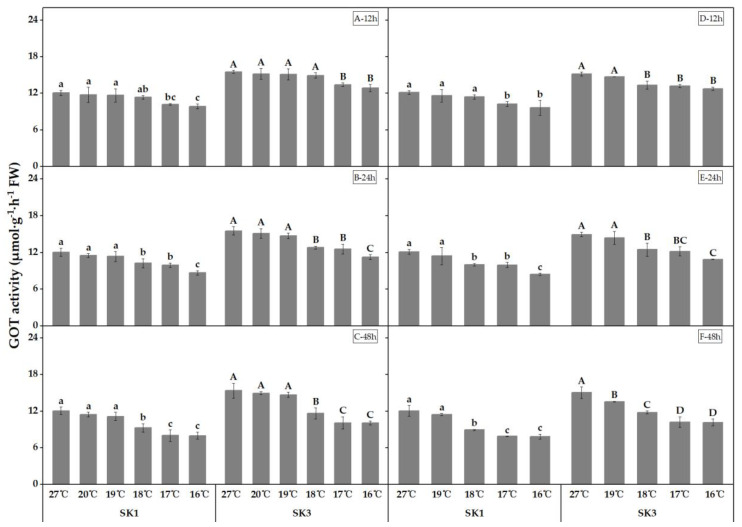
Effect of low-temperature stress on GOT activity in boll shells in 2020 (A−12 h stress; B−24 h stress; and C−48 h stress) and 2021 (D−12 h stress; E−24 h stress; and F−48 h stress). Statistical analysis showed that there were significant differences between the treatments of the SK1 variety when represented by different lowercase letters, and between the treatments of the SK3 variety when represented by different capital letters (*p* < 0.05).

**Figure 4:**
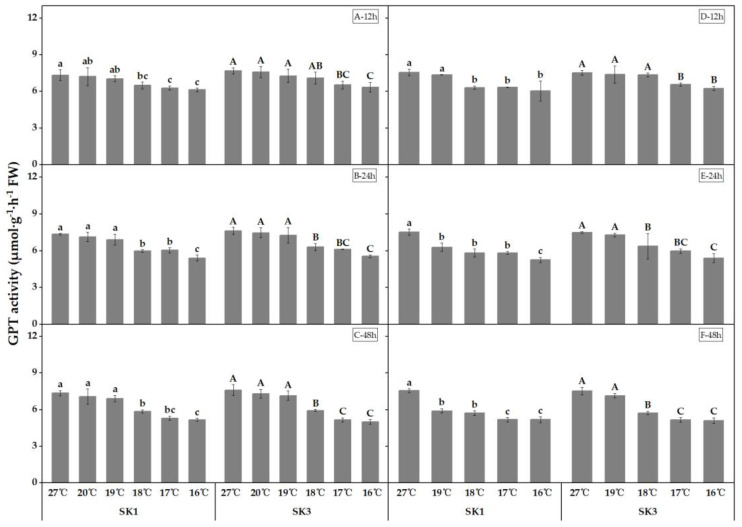
Effect of low-temperature stress on GPT activity in boll shells in 2020 (A−12 h stress; B−24 h stress; and C−48 h stress) and 2021 (D−12 h stress; E−24 h stress; and F−48 h stress). Statistical analysis showed that there were significant differences between the treatments of the SK1 variety when represented by different lowercase letters, and between the treatments of the SK3 variety when represented by different capital letters (*p* < 0.05).

The present study investigated the impact of low temperature on the activity of key enzymes involved in amino acid synthesis, namely GOT and GPT, in the boll shell. The results depicted in Figure 3 and Figure 4 demonstrate that the activities of both enzymes decreased as the temperature decreased. Furthermore, the study found that the threshold temperature required to cause a significant decrease in enzyme activity increased as the duration of low-temperature treatment was prolonged. Notably, extended exposure to low-temperature stress led not only to an elevation of the threshold temperature, but also to a further reduction in enzyme activity in the boll shell.

In 2020, exposure to low temperatures, for GOT activity in SK1 and SK3, for 12, 24, and 48 h resulted in threshold temperatures of 17 °C, 18 °C, and 18 °C, respectively. Similarly, in 2021, the threshold temperatures for GOT activity after 12, 24, and 48 h of low-temperature stress were 17 °C, 18 °C, and 18 °C in SK1, and 18 °C, 18 °C, and 19 °C in SK3. Compared to the control, a low temperature of 16 °C after 12 h of stress caused a decrease of 18.31% and 17.21% in GOT activity in SK1 and SK3, respectively. This reduction further increased to 28.08% and 27.50% after 24 h of stress, and to 33.75% and 34.66% after 48 h of stress in 2020. The results obtained in 2021 were consistent with those of 2020. These findings suggest that low-temperature stress has a significant negative impact on GOT activity in a boll shell, with a longer duration of stress leading to a higher threshold temperature for a significant reduction in activity.

In the year 2020, subjecting SK1 and SK3 to low-temperature treatment for durations of 12 h, 24 h, and 48 h revealed the low-temperature threshold for GPT activity to be 18 °C in SK1 and 17 °C, 18 °C, and 18 °C in SK3, respectively. Similarly, in 2021, the threshold temperatures for GPT activity following low-temperature stress for 12 h, 24 h, and 48 h were determined to be 18 °C, 19 °C, and 19 °C in SK1, and 17 °C, 18 °C, and 18 °C in SK3. Compared to the control, a low temperature of 16 °C after 12 h of stress caused a decrease of 16.39% and 17.52% in GPT activity in SK1 and SK3, respectively; this further increased to 26.47% and 27.10% after 24 h of stress, and to 29.61% and 34.18% after 48 h of stress in 2020, respectively. The results obtained in 2021 were consistent with those of 2020.

**Figure 5:**
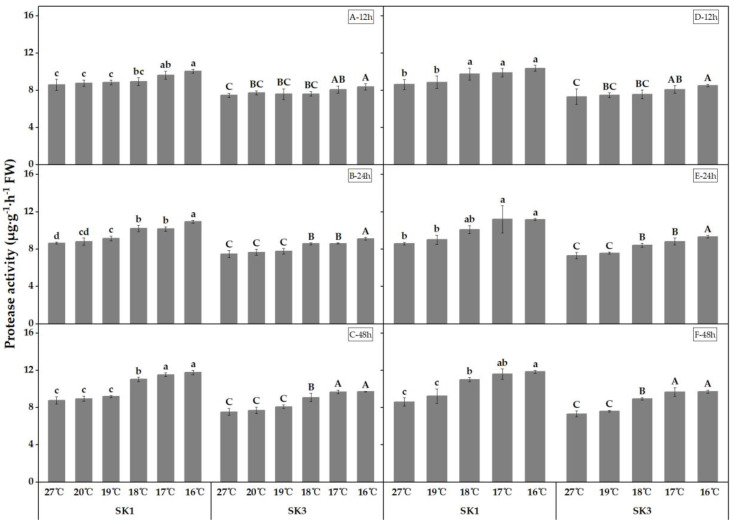
Effect of low-temperature stress on protease activity in boll shells in 2020 (A−12 h stress; B−24 h stress; and C−48 h stress) and 2021 (D−12 h stress; E−24 h stress; and F−48 h stress). Statistical analysis showed that there were significant differences between the treatments of the SK1 variety when represented by different lowercase letters, and between the treatments of the SK3 variety when represented by different capital letters (*p* < 0.05).

**Figure 6:**
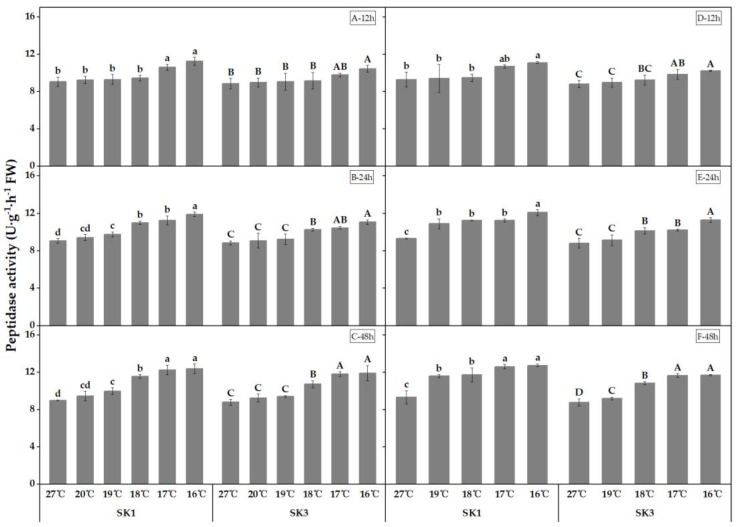
Effect of low-temperature stress on peptidase activity in boll shells in 2020 (A−12 h stress; B−24 h stress; and C−48 h stress) and 2021 (D−12 h stress; E−24 h stress; and F−48 h stress). Statistical analysis showed that there were significant differences between the treatments of the SK1 variety when represented by different lowercase letters, and between the treatments of the SK3 variety when represented by different capital letters (*p* < 0.05).

The activities of protease and peptidase, which are the enzymes responsible for protein degradation, were found to increase with decreasing temperature, as depicted in Figure 5 and Figure 6. Furthermore, the threshold temperature causing a significant increase in the activities of these enzymes in boll shell increased with prolonged low-temperature treatment.

In 2020 and 2021, we investigated the effect of low temperature on protease activity in the boll shell and found that prolonged exposure to low temperature increased the threshold temperature required for a significant increase in protease activity (Figure 5). Specifically, after 12, 24, and 48 h of low-temperature treatment in 2020, the threshold temperature for protease activity in SK1 was observed to be 17 °C, 19 °C, and 18 °C, respectively, while for SK3, it was 17 °C, 18 °C, and 18 °C, respectively. Similarly, in 2021, the threshold temperature for protease activity in SK1 was 18 °C, 17 °C, and 18 °C after 12, 24, and 48 h of low-temperature stress, respectively, while for SK3, it was 17 °C, 18 °C, and 18 °C after 24 h and 48 h of low-temperature stress. Notably, after 12 h of stress at a low temperature of 16 °C, protease activity decreased by 16.88% in SK1 and 12.27% in SK3 compared to the control. This reduction in protease activity further increased to 26.64% in SK1 and 22.14% in SK3 after 24 h of stress, and to 33.96% in SK1 and 28.77% in SK3 after 48 h of stress in 2020. Similar findings were observed in 2021. Our results suggest that the threshold temperature for a significant increase in protease activity in response to low-temperature stress increases with a longer exposure to low temperature, and protease activity in the boll shell is positively correlated with decreasing temperature.

In the year 2020, subjecting SK1 and SK3 to low-temperature treatment for durations of 12 h, 24 h, and 48 h resulted in critical low temperatures of 17 °C, 19 °C, and 19 °C for SK1, and 16 °C, 18 °C, and 18 °C for SK3 in relation to peptidase activity, as depicted in Figure 6. Subsequently, in 2021, the threshold temperature for peptidase activity was determined to be 16 °C, 19 °C, and 19 °C following low-temperature stress of 12 h, 24 h, and 48 h in SK1, as well as 17 °C, 18 °C, and 19 °C following low-temperature stress of 24 h and 48 h in SK3. When compared to the control, exposure to 16 °C for 12 h caused a reduction of 24.29% in peptidase activity in SK1, and a reduction of 18.22% in SK3. Furthermore, after 24 h of stress, peptidase activity decreased by 31.55% in SK1 and 24.97% in SK3. This reduction further declined to 37.63% in SK1 and 35.34% in SK3 after 48 h of stress in 2020. Comparable outcomes were observed in 2021.

### 2.3. Relationship between Boll Shell Bt Protein Content and Nitrogen Metabolism

**Figure 7:**
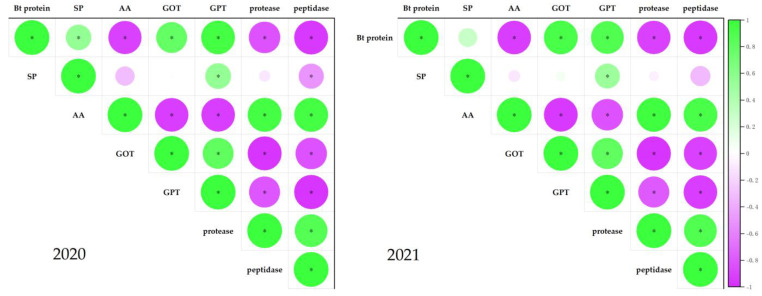
Correlations between Bt insecticidal protein-content- and nitrogen-metabolism-related parameters in 2020 and 2021. AA−free amino acid and SP−soluble protein. Asterisks indicate significance at the 0.05 (*) level.

The correlation analysis of Bt protein content with nitrogen metabolism and key enzyme activities in boll shells (Figure 7) showed that the Bt protein content in boll shells was positively correlated with soluble protein content, GOT activity, and GPT activity (R_2020_ = 0.55 *, 0.79 *, 0.95 *; R_2021_ = 0.30, 0.94 *, 0.89 *), which were negatively correlated with free amino acid content, protease, and peptidase activity (R_2020_ = −0.91 *, −0.83 *, −0.96 *; R_2021_ = −0.92 *, −0.91 *, −0.95 *), respectively. It can be seen that the change in Bt protein content in boll shells is closely related to the synthesis and decomposition process of nitrogen metabolism, which also indicates that the physiological change in boll nitrogen metabolism leads to changes in Bt protein content, thereby affecting insect resistance.

## 3. Discussion

### 3.1. Prolonging the Low-Temperature Stress Treatment Duration Further Reduced the Insecticidal Protein Content and Increased the Threshold Temperature

Zhou et al. [35] have observed that exposure to low temperatures, particularly at 16 °C, increases the susceptibility of young leaves of Bt cotton to cotton bollworms during the peak flowering and boll stages. Similarly, Zhang et al. [36] reported that low-temperature stress reduces the expression of insecticidal proteins in cotton leaves, especially during the peak boll stage. Notably, the impact of temperature on insecticidal protein content in leaves is greater than that of humidity, with low temperature having a more significant effect than high temperature. During the boll-setting stage, the Bt protein content in cotton leaves decreases more rapidly compared to the peak flowering stage [26]. However, studies on the effects of low-temperature stress on cotton have mainly focused on leaves, with limited exploration on the impact of varying degrees and durations of low temperatures on Bt protein content, particularly in reproductive organs. During the cotton square stage, we found that low temperature reduced the content of insecticidal proteins in cotton squares [31], but the insect resistance of the cotton boll is one of the important factors determining yield. The boll shell is also the primary area where cotton bollworms harm cotton bolls. Therefore, the changes in insect resistance of boll shells under low temperature stress have more practical significance for agricultural production.

This study revealed that low-temperature stress has a significant impact on the levels of insecticidal proteins in the boll shell of Bt cotton, resulting in a substantial reduction. Additionally, as the duration of the stress increases, the threshold temperature causing a significant reduction in the insecticidal protein content also increases, exacerbating the reduction. In summary, low-temperature treatment negatively affects the expression of insecticidal protein in the boll shell of Bt cotton. The temperature threshold causing a significant reduction in the insecticidal protein content was found to be 17 °C after 12 h of low-temperature exposure, 18 °C after 24 h, and 20 °C after 48 h. Therefore, it is crucial to remain alert to low-temperature disasters that last more than 12 h and that drop below 17–20 °C during the boll stage in Bt cotton production. Such disasters can trigger significant pest outbreaks, resulting in substantial economic losses.

### 3.2. The Influence of Low Temperature on Nitrogen Metabolism Resulted in a Decrease in the Contents of Bt Protein

**Figure 8:**
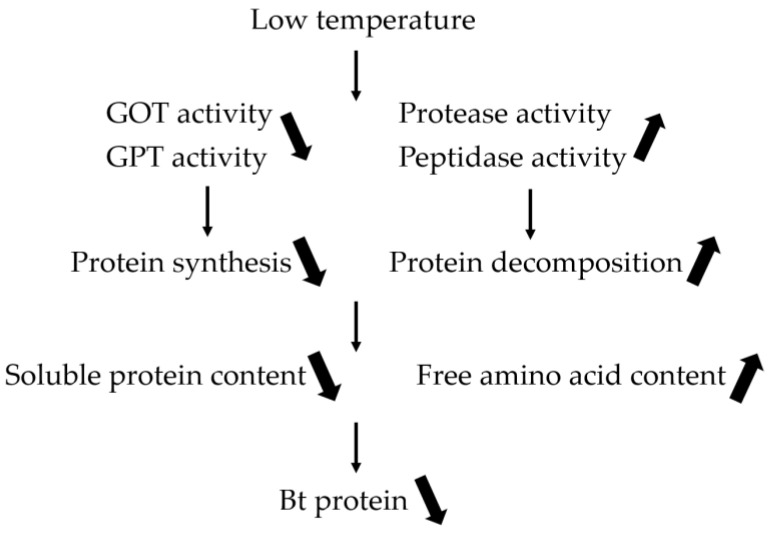
Low-temperature-affected Bt protein content through altered nitrogen metabolism.

Chen et al. [14,32,37,38,39,40,41] have previously reported on the strong link between the expression of insecticidal proteins and nitrogen metabolism in cotton. This study aims to investigate the effect of low-temperature stress on various physiological parameters of the boll shell in Bt cotton. Results show that under low-temperature stress, there was a significant decrease in soluble protein content, as well as reduced activity of glutamic oxaloacetic transaminase (GOT) and glutamic pyruvic transaminase (GPT). In contrast, free amino acid content, peptidase, and protease activities increased in response to low-temperature stress, with greater changes observed as temperature decreased and stress duration increased. Correlation analysis revealed a positive association between the Bt protein content in the boll shell and the soluble protein content, and GOT and GPT activities. However, a negative relationship with free amino acid content, protease, and peptidase activities was also found. These findings suggest that the observed reduction in insecticidal protein content in the boll shell under low-temperature stress was due to a decrease in protein synthesis ability and an increase in protein decomposition ability (Figure 8). Therefore, it is important to monitor changes in the insecticidal protein content of Bt cotton during the boll stage, which is vulnerable to low-temperature injury, and to take timely measures to improve nitrogen metabolism and mitigate the negative impact of low temperature and chilling injury on cotton production.

## 4. Materials and Methods

### 4.1. Experimental Materials and Design

The study was conducted during the 2020 and 2021 cotton growing seasons at Yangzhou University, Yangzhou, China (32°30′ N, 119°25′ E), using two Bt transgenic cotton cultivars, namely ‘Sikang1’ (conventional, SK1) and ‘Sikang3’ (hybrid, SK3). Seeds were sown in a greenhouse on April 15th in 2020 and April 18th in 2021. After 35 days, the resulting seedlings were transplanted to 50 cm high, 40 cm diameter pots with a 62.8 L volume. The pots were filled with sandy loam soil (Typic fluvaquents, Entisols) containing 18.8 g kg^−1^ organic matter and available N-P-K at 135.2, 22.8, and 80.9 mg kg^−1^, respectively. On May 17th, the day of transplanting, 1.5 g N as urea, 0.7 g P as single superphosphate, and 2.6 g as KCl were incorporated into the soil of each pot, and one seedling was transplanted per pot. The plants were watered daily, and at 46 days after transplanting, 1.6 g N as urea, 0.7 g P as single superphosphate, and 2.6 g as KCl were top-dressed into each pot. At 68 days after transplanting, 2.0 g N as urea was top-dressed into each pot.

This study was conducted using a completely randomized design with six replications, comprising six temperature regimes (optimum temperature: 27 °C and low temperatures: 16 °C, 17 °C, 18 °C, 19 °C, 20 °C) in 2020 and five temperature regimes (optimum temperature: 27 °C and low temperatures: 16 °C, 17 °C, 18 °C, 19 °C) in 2021. At the peak flowering stage (July 18th), flowers on the seventh to eighth fruiting branches were observed, and the temperature treatments began fifteen days after flower appearance by relocating the pots to environmentally controlled rooms with different temperature settings (14 h d^−1^ photoperiod at a photon flux density of 200 mmol m^−2^ s^−1^ and 70% relative humidity). Labeled bolls were collected at 12 h, 24 h, and 48 h after the treatments and stored at −80 °C for future measurements.

### 4.2. Data Collection

#### 4.2.1. The Bt Protein Content

The insecticidal protein contents of the cotton boll shells were determined with ELISA [42]. Three samples of boll shell tissue extracts were prepared by homogenizing the frozen tissue in 2 mL of extraction buffer (NaCl 8.0 g, KH_2_PO_4_ 0.2 g, and Na_2_HPO_4_ 2.96 g dissolved in 1000 mL of distilled water; 1 mL Tween). The centrifuge tube was shaken with hand, stored at 4 °C for 4 h, and then centrifuged at 1000× *g* for 15 min. The supernatant was the sample to be tested.

Quantification of the boll shell insecticidal protein was conducted using a commercially available kit (Scientific Service, Inc., China Agriculture University) according to Chen et al. [40]. Microtitration plates were coated with 100 μL of buffer solution (Na_2_CO_3_ 1.5 g, NaHCO_3_ 2.93 g dissolved in 1000 mL of distilled water, pH 9.6; 1 mL anti-rabbit immunoglobulin) and incubated at 37 °C for 4 h. The 50 μL antibodies, standard Cry1Ac insecticidal proteins, and samples were added to each well and incubated for further 30 min at 37 °C. Then, 100 μL of horseradish peroxidase-labeled goat anti-rabbit immunoglobulin was added to each well and incubated for 30 min at 37 °C. Finally, 100 μL of buffered enzyme substrate (o-Penylenediamine 0.2 g, C_6_H_8_O_7_·H_2_O 0.51 g, and Na_2_HPO_4_·12H_2_O 1.843 g dissolved in 100 mL distilled water, pH 5.0; 0.1 mL Tween; 40 μL 30% H_2_O_2_) was added, the enzyme reaction was carried out in the dark at 37 °C for 15 min, and then terminated using 50 μL of 2 mol·L^−1^ H_2_SO_4_. The absorbance was recorded at 490 nm.

#### 4.2.2. Soluble Protein and Amino Acid Content

The boll shell samples from different treatments were used for soluble protein content analysis. The samples were homogenized at 4 °C in 1.5 mL of cold water and centrifuged at 7100× *g* for 10 min. The supernatant was the sample to be tested. The total soluble protein content was determined by the Coomassie blue dye-binding assay of Zou [43]. Three samples of boll shell tissue extracts were prepared by homogenizing the frozen tissue in 1 mL of extracting solution (10% CH_3_COOH). The samples were centrifuged at 1000× *g* for 5 min. The supernatant was used for the analysis of amino acid concentration. The total free amino acid content was determined by ninhydrin assay [43].

#### 4.2.3. Glutamate Oxaloacetate Transaminase and Glutamic Pyruvic Transaminase Assay

The boll shell samples were homogenized in 2 mL of 0.05 mol·L^−1^ Tris-HCl extraction buffer (6.05 g Tris and 22.1 mL 2 mol·L^−1^ HCl dissolved in 1000 mL of distilled water, pH 7.2), and the homogenate was centrifuged at 20,000× *g* for 20 min at 4 °C. The supernatant was analyzed for GOT and GPT activity. The experiment was conducted according to Wu et al. [44].

A mixture of 0.5 mL of substrate solution (200 mmol L^−1^ DL-aspartic acid, 2 mmol L^−1^ α-ketoglutaric, pH 7.4) and 0.1 mL of the enzyme preparation was incubated at 37 °C for 1 h and then terminated using 0.5 mL of 1 mmol L^−1^ 2,4-dinitrophenylhydrazine. The reaction mixture was incubated for further 20 min at 37 °C and then 5.0 mL of 4 mol L^−1^ NaOH was added. The absorbance was recorded at 500 nm. The GOT activity, in terms of pyruvate production, was calculated from authentic pyruvate standards run simultaneously.

A mixture of 0.5 mL of substrate solution (200 mmol L^−1^ alanine, 2 mmol L^−1^ α-ketoglutaric, pH 7.4) and 0.1 mL of the enzyme preparation was incubated at 37 °C for 30 min, and then terminated using 0.5 mL of 1 mmol·L^−1^ 2,4-dinitrophenylhydrazine. The reaction mixture was incubated for a further 20 min at 37 °C and then 5.0 mL of 4 mol·L^−1^ NaOH was added. The absorbance was recorded at 500 nm. The GPT activity, in terms of pyruvate production, was calculated from authentic pyruvate standards run simultaneously.

#### 4.2.4. Assay of Protease and Peptidase Activity

The boll shell samples were homogenized in 1.5 mL of water and the homogenate was shaken for 30 min in a 40 °C water bath. Then, the homogenate was centrifuged at 14,200× *g* for 10 min at 10 °C. Protease activity was determined by the Folin–Ciocalteu assay of Dong et al. [42]. A mixture of a 0.25 mL of substrate solution (2% casein, pH 7.0) and 0.25 mL of the enzyme preparation was incubated at 40 °C for 10 min, and then terminated using 0.5 mL of 0.4 mol·L^−1^ TCA (trichloroacetic acid). The reaction mixture was incubated for a further 10 min at 40 °C and then centrifuged at 1000× *g* for 2 min. Finally, 0.5 mL of supernatant, 2.5 mL of 0.4 mol·L^−1^ Na_2_CO_3_, and 0.5 mL of 25% Folin–Ciocslteu were added to the centrifuge tubes and incubated for 20 min at 40 °C. The absorbance was recorded at 660 nm. The protease activity, in terms of tyrosine production, was calculated from the authentic tyrosine standards being run simultaneously.

The boll shell samples were homogenized at 4 °C in 1.5 mL of 5 mmol·L^−1^ Hepes extraction buffer (2.383 g Hepes, 0.7445 g EDTA-Na_2_, 0.617 g DTT, and 20 g PVP dissolved in 2000 mL of distilled water, pH 8.0) and then centrifuged at 15,000× *g* for 20 min at 4 °C. The supernatant was used to estimate the peptidase activity. The analysis was conducted according to Sun et al. [45]. A mixture of 0.1 mL of substrate solution (2% bovine hemoglobin, pH 5.2), 0.7 mL of 200 mmol·L^−1^ citric acid buffer, and 0.2 mL of the enzyme preparation was incubated at 38 °C for 1 h, and then terminated using 0.8 mL of 12% TCA. The reaction mixture was incubated for a further 30 min at 4 °C and then centrifuged at 4000× *g* for 2 min. The supernatant was used for the analysis of the amino acid content, which was determined by ninhydrin assay [43]. The absorbance was recorded at 570 nm.

### 4.3. Statistical Analysis

The statistical significance of the means was evaluated using the analysis of variance (ANOVA) method, and was followed by multiple mean comparisons utilizing the least significant difference (LSD) method at a significance level of 0.05. SAS 9.4 (SAS Institute, Cary, NC, USA) software was employed for these analyses. Pearson’s correlation analysis was used to investigate the relationships between the variables. Origin 2023 and Microsoft Excel 2019 were employed to create the figures and tables.

## 5. Conclusions

This study demonstrated that low-temperature stress has a significant impact on the insecticidal protein contents in the boll shell of Bt cotton. The threshold temperature leading to a substantial reduction in the protein content was found to increase with prolonged stress duration, resulting in a greater reduction. The investigation of nitrogen metabolism unveiled a reduction in the soluble protein content, as well as in the GPT and GOT activities, while free amino acid levels, peptidase, and protease activities increased in response to low-temperature stress. Furthermore, the results revealed a significant association between the Bt protein content in the boll shell, as well as decreased protein synthesis ability and increased proteolysis ability. Based on these observations, it is apparent that the reduction in Bt protein content under low-temperature stress could be attributed to a combination of elevated protein decomposition and reduced protein synthesis.

## Figures and Tables

**Table 1:** Effect of low-temperature stress on Bt insecticidal protein content in boll shell in 2020 and 2021 (ng g^−1^ FW).

			SK1			SK3	
Year	Temperature	12 h	24 h	48 h	12 h	24 h	48 h
2020	27 °C	129.68 ± 3.93 a	130.08 ± 9.98 a	130.01 ± 3.05 a	134.60 ± 2.02 A	134.10 ± 6.96 A	133.87 ± 2.53 A
	20 °C	128.53 ± 10.04 a	124.68 ± 7.30 a	119.97 ± 3.76 b	133.96 ± 3.70 A	129.25 ± 8.78 A	122.73 ± 6.51 B
	19 °C	128.76 ± 9.73 a	124.21 ± 1.91 a	118.88 ± 8.22 b	133.54 ± 2.10 A	126.55 ± 2.15 A	122.87 ± 5.17 B
	18 °C	128.25 ± 2.93 a	111.41 ± 3.14 b	107.16 ± 6.38 c	129.79 ± 1.42 AB	112.71 ± 3.01 B	107.30 ± 2.89 C
	17 °C	115.18 ± 1.24 b	109.90 ± 1.44 b	95.30 ± 3.80 d	125.12 ± 2.76 B	111.46 ± 3.74 BC	97.42 ± 1.97 D
	16 °C	116.08 ± 3.54 b	99.08 ± 3.23 c	94.05 ± 5.72 d	125.37 ± 3.11 B	102.46 ± 5.41 C	96.14 ± 6.13 D
2021	27 °C	120.43 ± 4.46 a	120.82 ± 4.18 a	117.93 ± 3.68 a	133.10 ± 8.56 A	132.82 ± 8.69 A	132.12 ± 8.59 A
	19 °C	118.88 ± 1.14 a	116.66 ± 8.27 a	107.88 ± 1.53 b	132.52 ± 5.20 A	128.69 ± 4.52 A	121.17 ± 10.12 A
	18 °C	118.00 ± 0.73 a	106.90 ± 2.72 b	95.74 ± 4.25 c	130.73 ± 3.54 AB	119.00 ± 1.00 B	107.83 ± 0.95 B
	17 °C	111.76 ± 1.32 b	102.46 ± 6.52 b	84.20 ± 0.76 d	124.68 ± 0.35 B	113.90 ± 3.20 B	95.31 ± 2.51 C
	16 °C	111.35 ± 4.61 b	90.72 ± 1.18 c	83.55 ± 2.56 d	124.44 ± 1.95 B	101.08 ± 4.29 C	94.73 ± 2.22 C

Note: FW represents fresh weight. Statistical analysis showed that there were significant differences between the treatments of the SK1 variety when represented by different lowercase letters, and between the treatments of the SK3 variety when represented by different capital letters (*p* < 0.05).

## Data Availability

The data presented in this study are available on request from the corresponding author.
